# Novel Nanoliposomes Alleviate Contrast-Induced Nephropathy by Mediating Apoptosis Response in New Zealand Rabbits

**DOI:** 10.3389/fmolb.2021.681849

**Published:** 2021-07-06

**Authors:** Peng Zhang, Xue Zhang, Jing Zhang, Yanqiu Song, Ting Liu, Zhican Zeng, Xiaofeng Fu, Han Fu, Hong Zhang, Qin Qin, Naikuan Fu, Zhigang Guo

**Affiliations:** ^1^ Department of Cardiology, Tianjin Chest Hospital, Tianjin, China; ^2^ Department of Cardiology, The Third Central Hospital of Tianjin, Tianjin, China; ^3^ Institute of Cardiovascular Disease, Tianjin Chest Hospital, Tianjin, China; ^4^ Graduate School of Tianjin Medical University, Tianjin, China

**Keywords:** nanoliposome, contrast-induced nephropathy, apoptosis, acute kidney injury, New Zealand rabbit

## Abstract

The aim of this study was to test the preventive effects of nano liposomes against contrast-induced nephropathy (CIN) in New Zealand rabbits. Sixty New Zealand rabbits were randomly divided into four groups, with 15 rabbits in each group: control group, contrast group, hydration group and nano liposome group. Serum creatinine (Scr) and Blood Urea Nitrogen (BUN) were measured before and after injection of the contrast agent iopromide. Oxidative stress markers, such as superoxide dismutase (SOD) and malondialdehyde (MDA), and apoptosis markers, such as Bcl2-Associated X (Bax) and B-cell lymphoma-2 (Bcl-2), were measured by enzyme-linked immunosorbent assay (ELISA). Rabbits were killed 24 h after injection of the contrast medium and both kidneys were removed. Real-time Polymerase Chain Reaction (RT-PCR) and Western blot assays were performed in kidney tissue. Pathological changes were analyzed under the optical and electron microscope. Compared with the hydration group, the nano liposome group showed improved protection of renal function, with significantly different Scr and BUN levels, incidence of CIN, apoptosis index, RT-PCR and Western blot protein expression patterns. Under the optical and electron microscope, the renal injury in the nano liposome group was less than in the hydration group. However, based on SOD and MDA, there was no significant difference in oxidative stress when compared with the hydration group. Apoptosis is an important mechanism in CIN. Nano liposomes can prevent the occurrence of CIN by decreasing apoptosis, reducing damage to the kidney by the contrast agent.

## Introduction

Contrast-induced nephropathy (CIN) is an increase in serum creatinine (Scr) value by more than 25% from baseline or of the absolute value by more than 44.2 μmol/L within 24–72 h of contrast agent injection, excluding other factors affecting renal function (Rihal et al., 2002). With the rapid development of interventional diagnostic tests and treatments, the use of contrast media and the incidence rate of CIN are increasing year by year. It has been reported that CIN has become the third most frequent cause of hospital-acquired acute kidney injury, after surgical procedures and drugs, accounting for about 10% of the total (Toprak, 2007). Data show that when CIN occurs after percutaneous coronary intervention (PCI), the incidence of cardiovascular adverse events is significantly higher than in patients without CIN, even if renal function recovers. Chinese and international scholars currently view CIN after PCI as an independent predictor of cardiovascular adverse events. The incidence rate of CIN increases significantly, by as much as 50%, when other risk factors are present, such as diabetes and old age. Unfortunately there are no specific, effective treatments for CIN, so the key is to adopt preventive measures (Chen et al., 2014).

Although contrast medium is constantly being improved, the incidence of CIN is still increasing and the mortality rate is also on the rise. Until now, the pathogenic mechanisms underlying CIN have not been fully elucidated. It is speculated that they may be related to the combined effects of contrast medium-induced renal medullary vascular relaxation and contraction imbalance, renal tubular ischemia, direct toxicity of the contrast media against the kidney, oxidative stress, inflammatory response, apoptosis, etc., (Heyman et al., 2010; Geenen et al., 2013; Jorgensen, 2013).

A nano liposome is one type of lipid micro-capsular structure (Carr et al., 2008). In liposomes the particle size is far smaller than that of ordinary lipids. Liposomes show a high degree of self-deformability, and their structure includes an aqueous core. The stability, conductivity, conduction and adhesion properties of nano liposomes in cell fluid, plasma and other bodily fluids show great advantages over ordinary liposomes (Goldberg and Klein, 2012). In addition, nano liposomes, like human fatty acids, can quickly enter the renal cell tissue through the intercellular space, and then quickly and efficiently enter the organelles inside the kidney cells (Faulk et al., 2013; Yamada and Harashima, 2015). Nano liposomes are not biologically toxic and do not produce allergic reactions (Khalil et al., 2008).

Many theories or molecular mechanisms of action have been proposed to explain why nano liposomes relieve or reduce nephrotoxicity caused by contrast agents; in particular, the “metabolic theory” and the “solubilization effect mechanism” (Ueno, 2011). The metabolic theory posits that nano liposomes can enhance the free fatty acid content of kidney cells, effectively providing energy for renal cell metabolism and physiological functions; nano liposomes can stabilize the mitochondrial membrane potential and reverse the inhibitory effect of contrast medium on normal physiological processes (Ibarguren et al., 2013). According to the solubilization effect mechanism, nano liposomes can effectively enhance the fatty acid content and fuse directly with the membrane of organelles in renal cells, increasing the surface area and stabilizing the renal cell membrane (Bertrand et al., 2010).

After reviewing the scientific literature, we realized there were no studies on the effects and mechanisms of action of nano liposomes against CIN, either in China or abroad. Therefore, we decided to study whether nano liposomes could be used to prevent or treat CIN, and to explore the possible mechanisms of action. The ultimate aim is to reduce the nephrotoxic effects of contrast agents, reduce the hospitalization time, lower the hospitalization expenses, reduce cardiovascular and renal events after PCI, and improve the short- and long-term prognosis of patients.

## Materials and Methods

### Experimental Design and Grouping

A total of 60 New Zealand rabbits were used for all experiments. The animals were obtained from the Beijing huafukang Biotechnology Co., Ltd. and kept in a cage with access to food and water ad libitum (12 h light/dark cycle) at 25 ± 2°C and 45–50% humidity. 60 New Zealand rabbits (Bodyweight: 2.0–3.0 kg, male or female) were randomly divided into four groups, with 15 rabbits in each group. Group A (control group): intravenous injection of normal saline (24 ml/kg, from Shanghai Baite Pharmaceutical Co., Ltd, China); Group B (contrast group): intravenous injection of contrast agent iopromide injection (24 ml/kg, from Bayer healthcare Co., Ltd, Germany); Group C (hydration group): Intravenous injection of the contrast agent iopromide injection (24 ml/kg) and intravenous injection of normal saline (24 ml/kg); Group D (nano liposome group): Intravenous injection of contrast agent iopromide injection (24 ml/kg) and infusion of nano liposomes (2 ml/kg, Shanghai Shunna Technology Co., Ltd., China). We performed the following experiments on all rabbits after 4 weeks of treatment. Animal protocols adopted in the present study were approved by the medical ethics committee of Tianjin Chest Hospital (Approval No. 2020 YS-047-01), which follows the guidelines established by the United States National Institutes of Health.

### Operation Steps of Animal Experiment

1) Anesthesia and fixation: the rabbits were anesthetized by intra-peritoneal injection of 3% Pentobarbital injection (Shanghai Shangyao Xinya Pharmaceutical Co., Ltd., Shanghai, China); after the corneal reflex and pain reflex disappeared, New Zealand rabbit was fixed on the animal operating frame, and the skin of both ears was routinely prepared and shaved. 2) Wiped the skin on the surface of vein with alcohol cotton swab, pressed the root of auricular vein or used sterile forceps to ligate the root of auricular vein if necessary, inserted indwelling needle rapidly along ear vein, drew 2 ml blood from indwelling needle with 5 ml empty needle, put into the prepared biochemical coagulation promoting tube (Cangzhou Yongkang Medical Products Co., Ltd., Hebei, China) which had been disinfected, and marked the group and rabbit number in advance. 3) Contrast agent, saline or nano liposomes were injected into the ear vein along the indwelling needle at the prescribed time. 4) Compressed ear vein to stop bleeding. 5) the blood extracted was centrifuged by high-speed centrifuge (Thermo Fisher Scientific Co., Ltd., Shanghai, China) and the serum was collected. CIN was defined as a 25% increase in Scr within 24 h after injection.

24 h after the injection of contrast medium, New Zealand rabbits were killed, and the left kidney was cut obliquely outward and downward about 1.5–2 cm. The left kidney was separated and exposed; the fat tissue and renal capsule around the left kidney were stripped, and the renal pedicle was exposed. Subsequently, the left kidney was removed along the upper part of the vascular clamp. The cortices were washed with 4°C buffer solution. Then they were put into containers containing formalin (Guangdong Kangnaixin Biomedical Technology Co., Ltd., Guangdong, China) and 4°C stationary solution (2.5% glutaraldehyde, Qingdao jieshikang Biotechnology Co., Ltd., Shandong, China), respectively. The specimens were immediately put into the container containing 4°C solid solution for sealing. At the same time, the appropriate amount of left renal cortices were washed and dried with saline, weighed, and stored at −80°C until further processing. In the same way, the right kidney was exposed, and the renal tissue was taken for reserve.

### Detection Index

**FIGURE 1:**
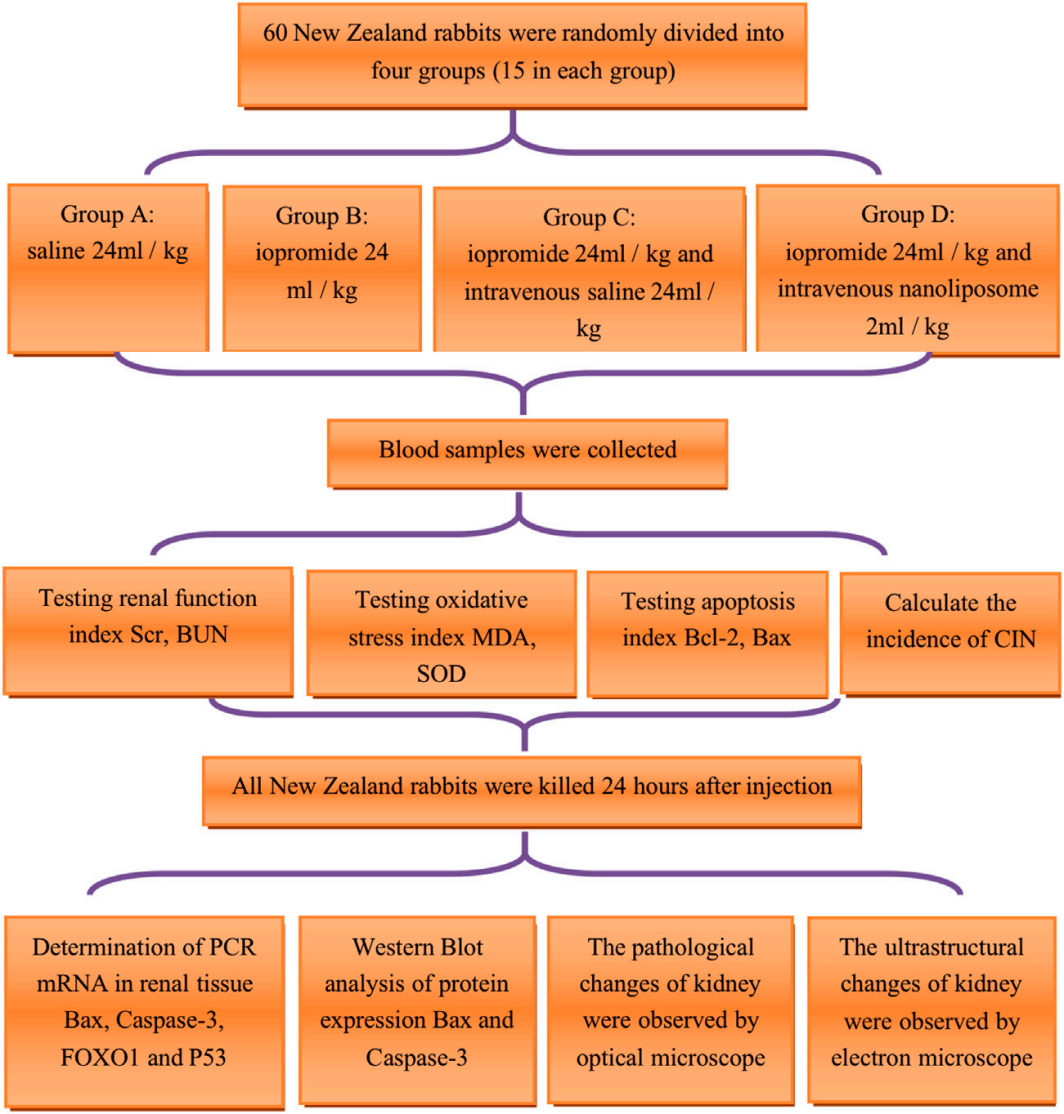
Technology roadmap of animal experiment. Scr = Serum creatinine; BUN = blood urea nitrogen; MDA = malondialdehyde; SOD = superoxide dismutase; Bcl-2 = B-cell lymphoma/leukemia 2; Bax: Bcl-2 subfamily, pro-apoptotic factor; CIN=contrast Induced Nephropathy; Caspase-3=cysteine aspartate specific protease 3; FOXO1= Forkhead box O1; P53: a apoptosis inducing factor.

Scr, BUN, apoptosis suppressor gene Bcl-2, apoptosis promoting gene Bax, superoxide dismutase (SOD), malondialdehyde (MDA) were detected before and 2, 8, and 24 h after the injection of contrast medium. All New Zealand rabbits were killed, and the bilateral kidneys were removed 24 h after the injection of contrast medium. The expression of PCR mRNA such as Bax, Cysteine aspartate specific protease 3 (Caspase-3), Forkhead box O1 (FOXO1), and P53 in kidney tissue was determined; the western blotting was used to detect the protein expression of apoptosis promoting factors Bax and Caspase-3. The renal tissue and pathological ultra structural specimens of New Zealand rabbits were observed under optical microscope and electron microscope. The process flow chart was shown in Figure 1.

### Blood Biochemical Test

Blood samples were collected from the ear vein before and 2, 8, 24 h after injection of contrast medium. The blood samples were centrifuged and the serum was placed in the refrigerator at—20°C. Scr and BUN were detected by TBA-120FR automatic biochemical analyzer (Toshiba Corporation of Japan, Tokyo, Japan). Scr was measured using an enzymatic colorimetry, and BUN was determined by rate method (chromogenic urease method).

### Serum Enzyme-Linked Immuno Sorbent Assay Detection

The contents of Bcl-2, Bax, SOD and MDA were detected by enzyme-linked immuno sorbent assay (ELISA). The kit was provided by the elixir pharmaceutical company of Canada (Elixir Canada Medicine Company Ltd.). The procedure of ELISA was as follows: dispensed 100 μl of standards, specimens and controls into appropriate wells, dispensed 50 μl of Enzyme Conjugate into each well, incubated at thermostat 37°C for 60 min; rinsed and flicked the micro wells three times with 300 μl diluted wash concentrate; dispensed 50 μl of Chromogen A and Chromogen B reagent into each well, mixed for 5 s, incubated at 37°C in the dark for 20 min; stopped the reaction by adding 50 μl of stop solution to each well, read absorbance at 450 nm with a micro plate reader within 10 min (using multiskan MK3 system, Thermo Electron Corporation, Massachusetts, United States).

### Comparison of the Incidence Rate of Contrast Induced Nephropathy in Each Group

According to the changes of Scr before and 24 h after drug injection, the diagnosis of CIN was defined as the increase of Scr by more than 25% compared with the original baseline level within 24 h after injection of contrast agent.

### Real-Time Polymerase Chain Reaction was Used to Detect the messanger-Ribo Nucleic Acid Relative Expression of the Target Gene

Total RNA extraction by Trizol method: the kidney tissues were removed from −80°C, and then 100 mg of tissue was cut into pieces and put into 1 ml Trizol (1% beta-mercaptoethanol, Shanghai Shenggong bioengineering technology service company) cracking medium, homogenizer homogenized for 20 s, on ice for 2 min, repeated twice, and then centrifuged at 10,000 rpm for 5 min; took the supernatant and transferred the supernatant to a new 1.5 ml centrifuge tube, added chloroform and 1/5 Trizol reagent (200 µl), centrifugated at 12,000 rpm at 4°C for 15 min; transferred the supernatant to a new 1.5 ml centrifuge tube, added the same volume of isopropanol (500 µl) to the supernatant, and mixed it upside down for five times, centrifugated at 12,000 rpm at 4°C for 10 min; discarded the supernatant, 75% ethanol 1 ml was added to the sediments, centrifuged at 12,000 rpm for 5 min. Kept the sediments dry, the sediments were dissolved in 25 μl diethyl pyro carbonate (DEPC) treated water. The concentration of RNA was detected using the epoch system (BioTek Instrument Corporation, Vermont, United States).

Reverse transcription of RNA into complementary DNA (cDNA) (Revert cDNA synthesis kit, Fermentas, K1622) (using Eppendorf Mastercycler PCR system, Eppendorf AG, Hamburg, Germany): took 2 μg RNA, 1 μl random primer, made up the volume to 12 μl, 65°C 5 min, ice bath for 5 min; 5 × RT buffer 4 μl, 10 mmol/L deoxy-ribonucleoside triphosphate (dNTP) 2 μl, Moloney Murine Leukemia Virus (M-MuLV) 1 μl, ribonuclease (RNase) inhibitor 1 μl were added into the above reaction solution to make up the volume to 20 μl; 25°C 5 min, 42°C 60 min, 70°C 5 min. The cDNA products were stored at—20°C.

Relative expression of target gene mRNA (using 7,500 Real-time PCR system, Applied Biosystems Inc., California, United States): the gene sequence was searched in GenBank. According to the principle of primer design, the primer sequence was synthesized and entrusted to Beijing oak Dingsheng Biotechnology Co., Ltd (Table 1). SYBR Premix Ex Taq (Tli RNaseH Plus, Code No. RR420A) was a particular reagent for Real-time PCR by SYBR Green I chimeric fluorescence method. The Real-time PCR composition of the reaction system was shown in Table 2. The Real-time PCR reaction conditions: 95°C 30 s, 95°C 5 s, 60°C 34 s, 40 cycles. Real-time PCR reaction results: the relative mRNA expression was calculated by Ct value of target gene and GAPDH, it was expressed by 2^-△△Ct^. △△Ct (objective gene) = △Ct (objective gene)-△Ct (standard value); △Ct (objective gene) = Ct (objective gene)-Ct (housekeeping gene); △Ct (standard) = Ct (suppressor gene)-Ct (suppressor housekeeping genes).

**TABLE 1:** Real-time PCR primer sequence and annealing temperature.

Primer		Primer sequence	Fragment size (bp)	Annealing temperature (°C)	
Bax	Forward	5'--3′ TGG​GCT​GGA​CGC​TGG​ACT​TC	150	60
	Reverse	5'--3′ TGG​TGA​GTG​AGG​CGG​TGA​GC		
GAPDH	Forward	5’--3′CAA​GGC​TGT​GGG​CAA​GGT​CAT​C	110	60
	Reverse	5’--3′ TCT​CCA​GGC​GGC​AGG​TCA​G		
P53	Forward	5’--3′ATG​GAG​GAG​TCG​CAG​TCG​GAT​C	102	60
	Reverse	5’--3′GGT​GGT​CAG​CAG​GTT​GTT​CTC​AG		
Caspase-3	Forward	5’--3′CTA​AGC​CAC​GGT​GAT​GAA​GGA​GTC	142	60
	Reverse	5’--3′TGC​CTC​GGC​AAG​CCT​GAA​TAA​TG		
FOXO1	Forward	5’--3′CGC​ATG​ACC​CCA​GTG​AAG​ACA​TC	115	60
	Reverse	5’--3′CAC​CCA​TCC​TGC​CAT​AGC​CAT​TG		

Bax: Bcl-2 subfamily, pro-apoptotic factor; GAPDH = glyceraldehyde-3-phosphate dehydrogenase; P53: a apoptosis inducing factor; Caspase-3 = cysteine aspartate specific protease 3; FOXO1 = Forkhead box O1.

**TABLE 2:** Real-time PCR composition of reaction system.

Composition of reaction system	Sample volume (μl)
qPCR master mix	10.0
ROX	0.40
5 μmol/L forward primer	1.00
5 μmol/L reverse primer	1.00
cDNA	1.00
DDH_2_O	6.60
Total volume	20.00

qPCR Master Mix = Taq Polymerase Chain Reaction Master Mix; ROX = ROX Reference Dye; cDNA = complementary DNA; DDH_2_O = double distilled water.

### Western Blot Analysis

Western blotting was used to detect the protein expression of apoptosis promoting factors Bax and Caspase-3 in kidney tissues. The total protein was extracted from 100 mg New Zealand rabbit kidney tissue, and the protein concentration was determined by BCA (bicinchoninic acid) protein concentration detection kit (Solarbio, PC0020, Beijing Solarbio Technology Co., Ltd.). The protein samples (30 µg per well) were separated on 10% sodium dodecyl sulfate-polyacrylamide gel electrophoresis (SDS-PAGE) 80–100 voltage and transferred to polyvinylidene difluoride (PVDF) membranes (Merck Millipore Ltd., Darmstadt, Germany). Subsequently, the membranes were transferred to TBST (Tris-buffered saline and Tween 20) buffer containing 5% skimmed milk powder and were incubated for 2 h at 25°C. The membranes were washed three times in TBST for 5 min, and the samples were incubated overnight with primary antibodies at 4°C with gentle shaking. The primary antibodies included Bax (1:500, 21 kDa, Bioss), Caspase-3 (1:500, 28 kDa, Bioss), GAPDH (1:10,000, 37 kDa, Bioss). GAPDH was evaluated as a loading control. The secondary antibodies goat anti-rabbit IgG (1:5,000, Wuhan Boster Biological Technology., Ltd, Hubei, China) were incubated for 1.5 h the following morning, and the samples were washed with TBST three times for 10 min. After incubation, the color was started, and the ratio of the target protein to the gray value of internal reference GAPDH was calculated. It was regarded as its relative optical density value. The reactions were visualized using BIO RAD ChemiDoc XRS + Imaging System (BIO RAD life medicine Co., Ltd, Shanghai, China).

### Observation of Renal Tissue Under the Optical Microscope

The kidney specimens fixed in formalin were washed thoroughly for 2 h, dehydrated by 70, 80, 95, and 100% alcohol gradients successively. After being immersed in wax and embedded in paraffin, the specimens were sectioned continuously with a thickness of 5 µm and stained with Haematoxylin (HE). The pathological changes of renal tubules and other tissues were observed under the OLYMPUS optical microscope (Olympus (China) Investment Co., Ltd, Beijing, China).

### Observation of Renal Tissue Under the Electron Microscope

The prepared 1 mm^3^ renal tissue was cut into ultrathin sections of 50–70 nm after dehydration, immersion, embedding, and staining. The ultra structural changes of endothelial cells, mitochondria, and endoplasmic reticulum of the kidney were observed under the HITACHI H800 electron microscope (Hitachi group company, Tokyo, Japan). The results were recorded, the pictures with different multiples were selected and saved, and the electron microscope images were printed and preserved. The electron microscope examination was performed with the assistance of the Tianjin Institute of Biotechnology, Chinese Academy of Sciences.

### Statistical Analysis

IBM SPSS 25.0 statistical software was used for analysis. The experimental data was expressed as mean ± standard deviation ($\bar{x}$ ± s). If the data were normally distributed, one-way analysis of variance (ANOVA) was used to compare the four groups, and the LSD test (when the variance was homogeneous) or Dunnett’s *t* test (when the variance was not homogeneous) was used for the comparison between the two groups. Chi square test and Fisher exact test were used to analyze categorical variables, and the results were reported in figures and percentages. *p* < 0.05 was statistically significant.

## Results

The general characteristics of the four groups are presented in Table 3.

**TABLE 3:** Baseline characteristics and serum biochemical and oxidative stress apoptosis parameters of rabbits in the four groups.

Variables	Control group	Contrast group	Hydration group	Nano liposome group	*p* Value
Weight (kg)	2.38 ± 0.22	2.48 ± 0.25	2.32 ± 0.30	2.34 ± 0.33	0.407
TG (mmol/L)	0.68 ± 0.04	0.71 ± 0.10	0.68 ± 0.07	0.79 ± 0.16^∗##^	<0.05
TC (mmol/L)	1.29 ± 0.11	1.17 ± 0.13	1.21 ± 0.23	1.16 ± 0.17	0.127
HDL-c (mmol/L)	0.75 ± 0.13∗∗	0.95 ± 0.23	0.80 ± 0.20∗	0.74 ± 0.11^∗∗^	<0.01
LDL-c (mmol/L)	0.42 ± 0.71	0.42 ± 0.68	0.47 ± 0.12	0.38 ± 0.16	0.249
ALT (U/L)	27.33 ± 7.89	27.47 ± 5.03	29.60 ± 3.29	30.13 ± 2.12	0.319
AST (U/L)	16.45 ± 5.99	17.07 ± 3.71	17.73 ± 3.59	18.20 ± 2.60	0.680
Scr(umol/L)	79.13 ± 15.25	86.87 ± 28.98	87.07 ± 16.37	79.80 ± 17.10	0.561
BUN(mmol/L)	5.29 ± 1.77	6.15 ± 2.52	5.67 ± 2.29	4.87 ± 2.13	0.433
SOD (IU/mL)	1.60 ± 1.02	1.30 ± 0.89	1.66 ± 1.05	1.45 ± 0.90	0.753
MDA (nmol/L)	6.85 ± 5.09	4.62 ± 3.13	6.69 ± 6.44	8.78 ± 7.16	0.268
Bcl-2	1169.16 ± 238.74	921.87 ± 420.90	935.30 ± 570.83	1137.37 ± 215.98	0.353
Bax	484.88 ± 137.83	486.55 ± 203.17	391.20 ± 178.69	426.13 ± 111.45	0.300

Values are expressed as mean ± SD. TG = triglyceride; TC = cholesterol; HDL-c = high-density lipoprotein cholesterol; LDL-c = low-density lipoprotein cholesterol; ALT = Alanine aminotransferase; AST = Aspartate aminotransferase; Scr = Serum creatinine; BUN = blood urea nitrogen; SOD = superoxide dismutase; MDA = malondialdehyde; Bcl-2 = B-cell lymphoma/leukemia two; Bax: Bcl-2 subfamily, pro-apoptotic factor. ^∗^Compared with the contrast group, *p* < 0.05. ^∗∗^Compared with the contrast group, *p* < 0.01. ^#^Compared with the hydration group, *p* < 0.05. ^##^Compared with the hydration group, *p* < 0.01.

### Nano liposome Administration Decreases Serum Creatinine and Blood Urea Nitrogen Values and Improves Renal Function

**FIGURE 2:**
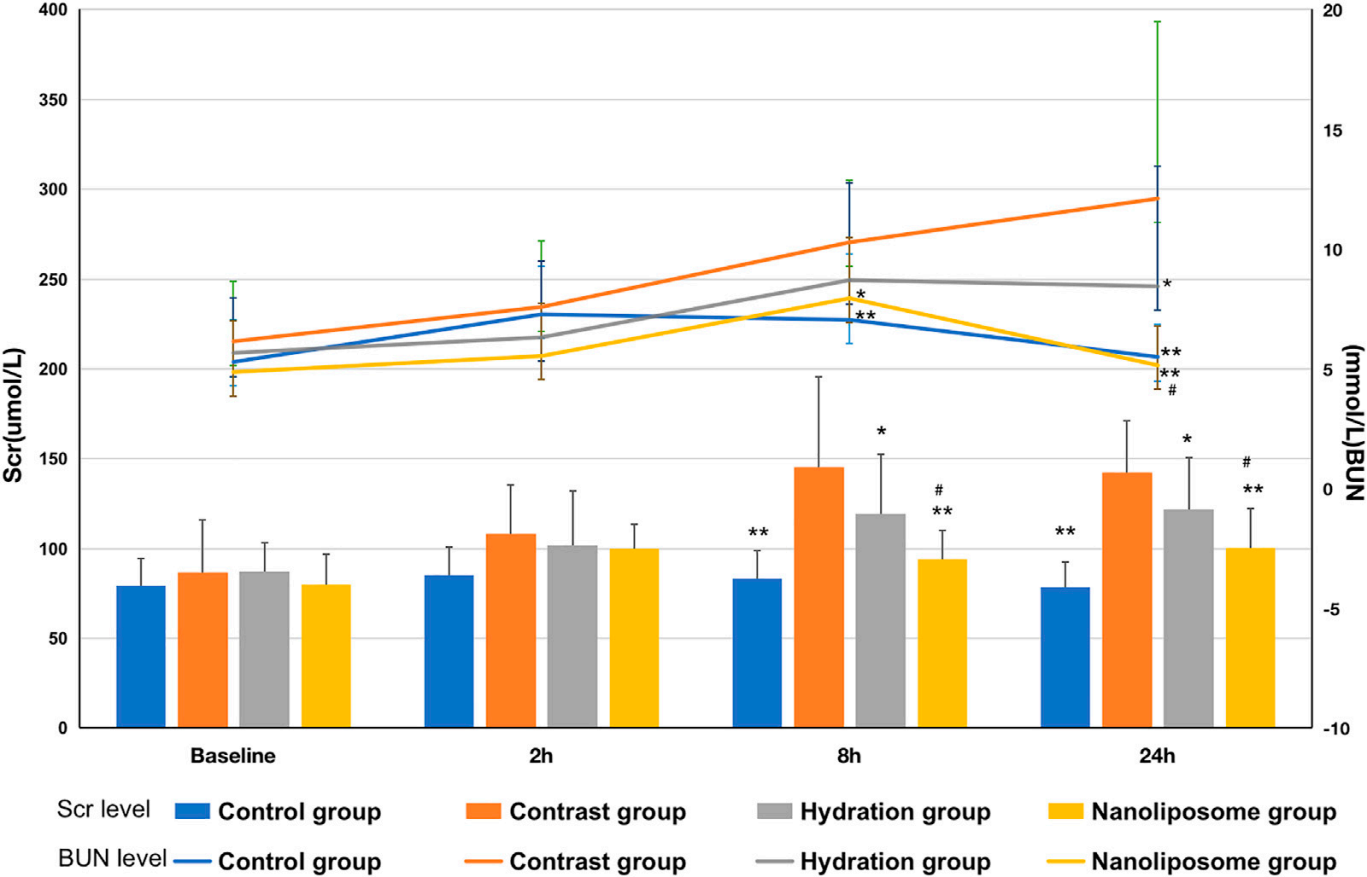
Comparison of Scr and BUN in four groups. Scr = Serum creatinine; BUN = blood urea nitrogen. Values are expressed as mean ± SD. *Compared with the contrast group, *p*<0.05. **Compared with the contrast group, *p*<0.01. ^#^Compared with the hydration group, *p*<0.05. ^##^Compared with the hydration group, *p*<0.01.

There were no significant differences in baseline Scr and BUN values between the four groups. Scr values were significantly higher than baseline in the four groups 8 and 24 h after injection of contrast medium. Similarly, BUN values also increased significantly in the four groups 8 and 24 h after injection of the contrast medium. Importantly, the Scr value in the contrast group was significantly higher than that in the hydration and nano liposome groups 8 h after injection of contrast medium (145.33 ± 50.40 vs. 119.27 ± 33.34, *p* < 0.05; 145.33 ± 50.40 vs. 93.86 ± 16.04, *p* < 0.05). Similarly, 24 h after injection, the Scr value in the contrast group was significantly higher than in the hydration and nano liposome groups (142.47 ± 28.63 vs. 121.67 ± 28.78, *p* < 0.05; 142.47 ± 28.63 vs. 100.27 ± 22.04, *p* < 0.05). However, Scr values in the nano liposome group were lower than in the hydration group 8 and 24 h after injection (93.86 ± 16.04 vs. 119.27 ± 33.34, *p* < 0.05; 100.27 ± 22.04 vs. 121.67 ± 28.78, *p* < 0.05). 8 h after injection, the BUN value in the contrast group was higher than in the nano liposome group (10.29 ± 2.60 vs. 7.95 ± 2.54, *p* < 0.05), and 24 h after injection the BUN value in the contrast group was significantly higher than in the hydration and nano liposome groups (12.11 ± 7.39 vs. 8.45 ± 5.00, *p* < 0.05; 12.11 ± 7.39 vs. 5.15 ± 1.63, *p* < 0.05). The BUN value in the nano liposome group was lower than in the hydration group at 24 h (5.15 ± 1.63 vs. 8.45 ± 5.00, *p* < 0.05) (Figure 2).

### Nano liposome Administration Increases Anti-Apoptotic Bcl-2 and Decreases Pro-Apoptotic Bax Levels

**FIGURE 3:**
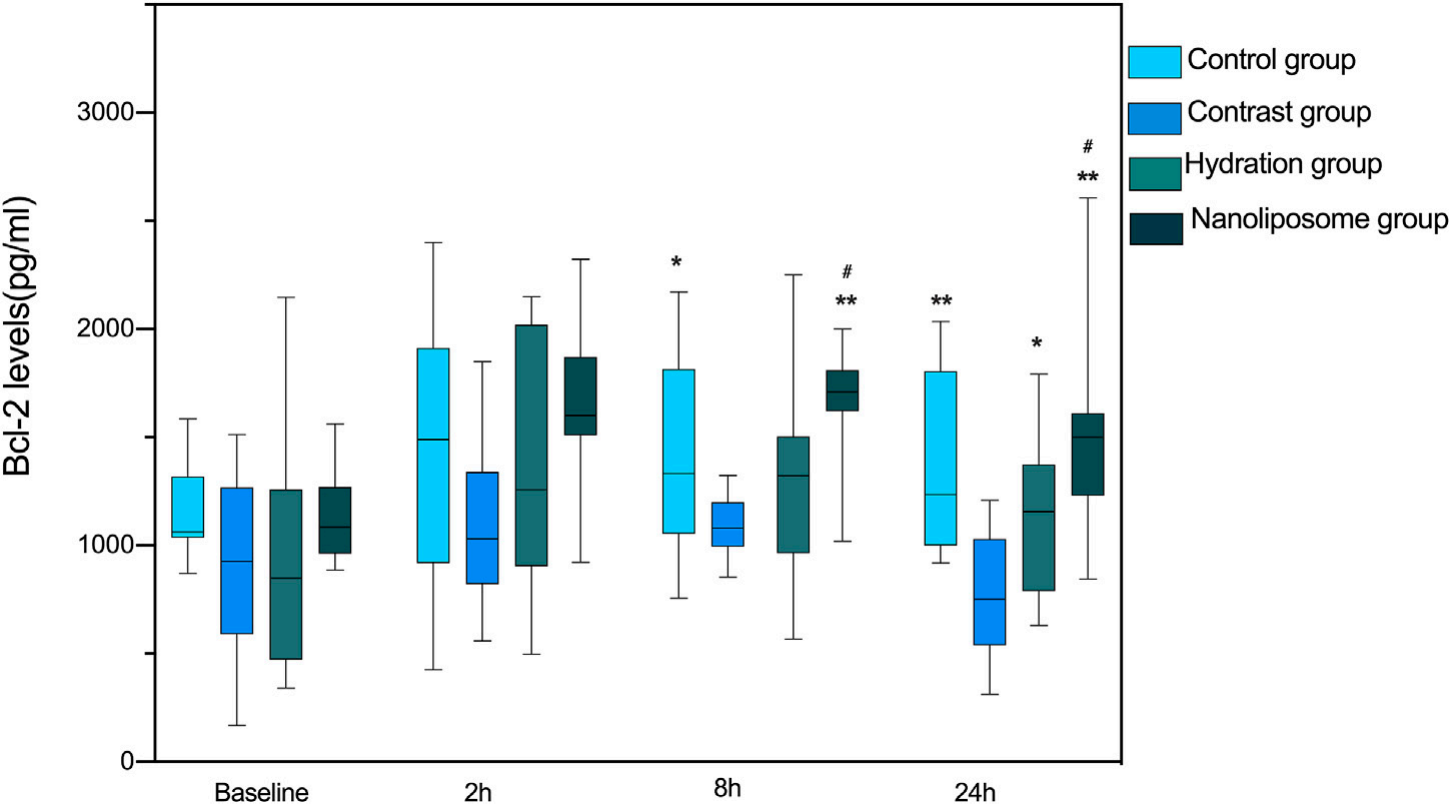
The contents of apoptosis index Bcl-2 in four groups were detected by ELISA. Bcl-2 = B-cell lymphoma/leukemia 2. Values are expressed as mean ± SD. *Compared with the contrast group, *p*<0.05. **Compared with the contrast group, *p*<0.01. ^#^Compared with the hydration group, *p*<0.05. ^##^Compared with the hydration group, *p*<0.01.

There were no significant differences in anti-apoptotic Bcl-2 levels at baseline between the four groups. However, there were significant differences in Bcl-2 serum levels between the four groups 8 and 24 h after injection of contrast medium (*p* < 0.05) (Figure 3). The levels of Bcl-2 in the nano liposome group were higher than in the contrast group at 8 and 24 h. Importantly, Bcl-2 levels in the nano liposome group were higher than in the hydration group at 8 and 24 h, and these differences were statistically significant (*p* < 0.05). In contrast, at the 2 h time point there were no statistically significant differences between groups (*p* > 0.05), although a trend could be seen.

**FIGURE 4:**
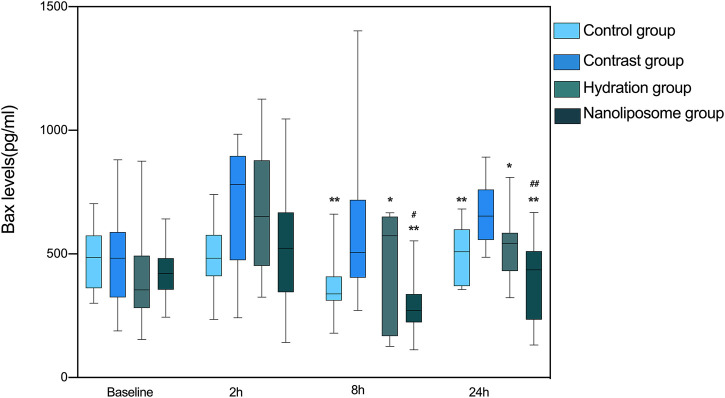
The contents of apoptosis index Bax in four groups were detected by ELISA. Bax: Bcl-2 subfamily, pro-apoptotic factor. Values are expressed as mean ± SD. *Compared with the contrast group, *p*<0.05. **Compared with the contrast group, *p*<0.01. ^#^Compared with the hydration group, *p*<0.05. ^##^Compared with the hydration group, *p*<0.01.

There were no significant differences in Bax levels between the four groups at baseline. However, Bax levels in the hydration and nano liposome groups were lower than in the contrast group at 8 and 24 h. Importantly, Bax levels in the nano liposome group were lower than in the hydration group at 8 and 24 h, and these differences were statistically significant (8 h *p* < 0.05, 24 h P < 0.01). At the 2 h time point there were no statistically significant differences between groups (*p* > 0.05), although a trend could be seen (Figure 4).

**FIGURE 5:**
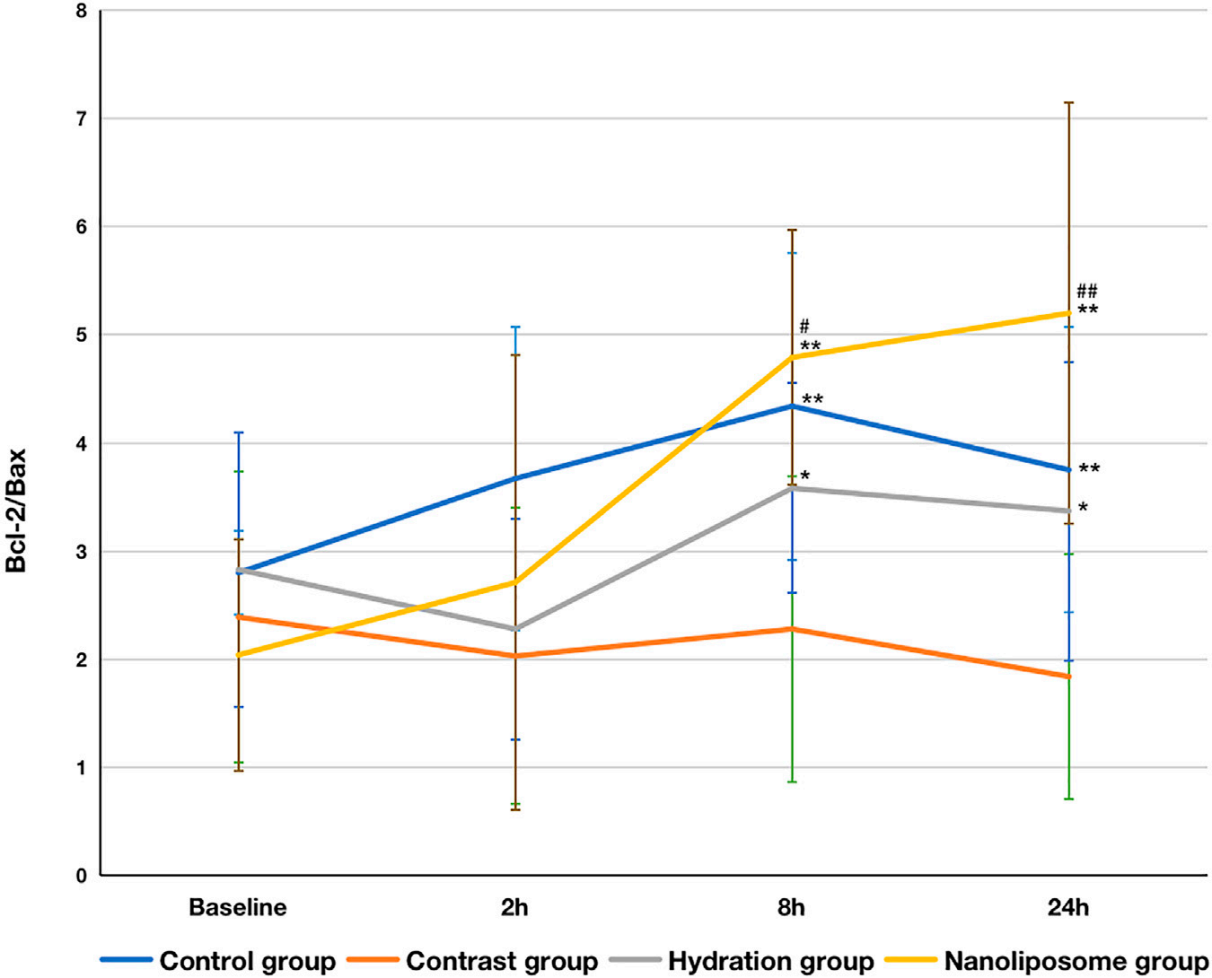
The contents of apoptosis index Bcl-2/Bax ratios in four groups were detected by ELISA. Bcl-2 = B-cell lymphoma/leukemia 2; Bax: Bcl-2 subfamily, pro-apoptotic factor. Values are expressed as mean ± SD. *Compared with the contrast group, *p*<0.05. **Compared with the contrast group, *p*<0.01. ^#^Compared with the hydration group, *p*<0.05. ^##^Compared with the hydration group, *P*<0.01.

In terms of the Bcl-2/Bax index, there were no significant differences between the four groups at baseline. However, the Bcl-2/Bax ratios in the hydration and nano liposome groups were higher than in the contrast group at 8 and 24 h. Importantly, the Bcl-2/Bax ratio in the nano liposome group was higher at 8 and 24 h than in the hydration group, and the differences were statistically significant (*p* < 0.05) (Figure 5).

**FIGURE 6:**
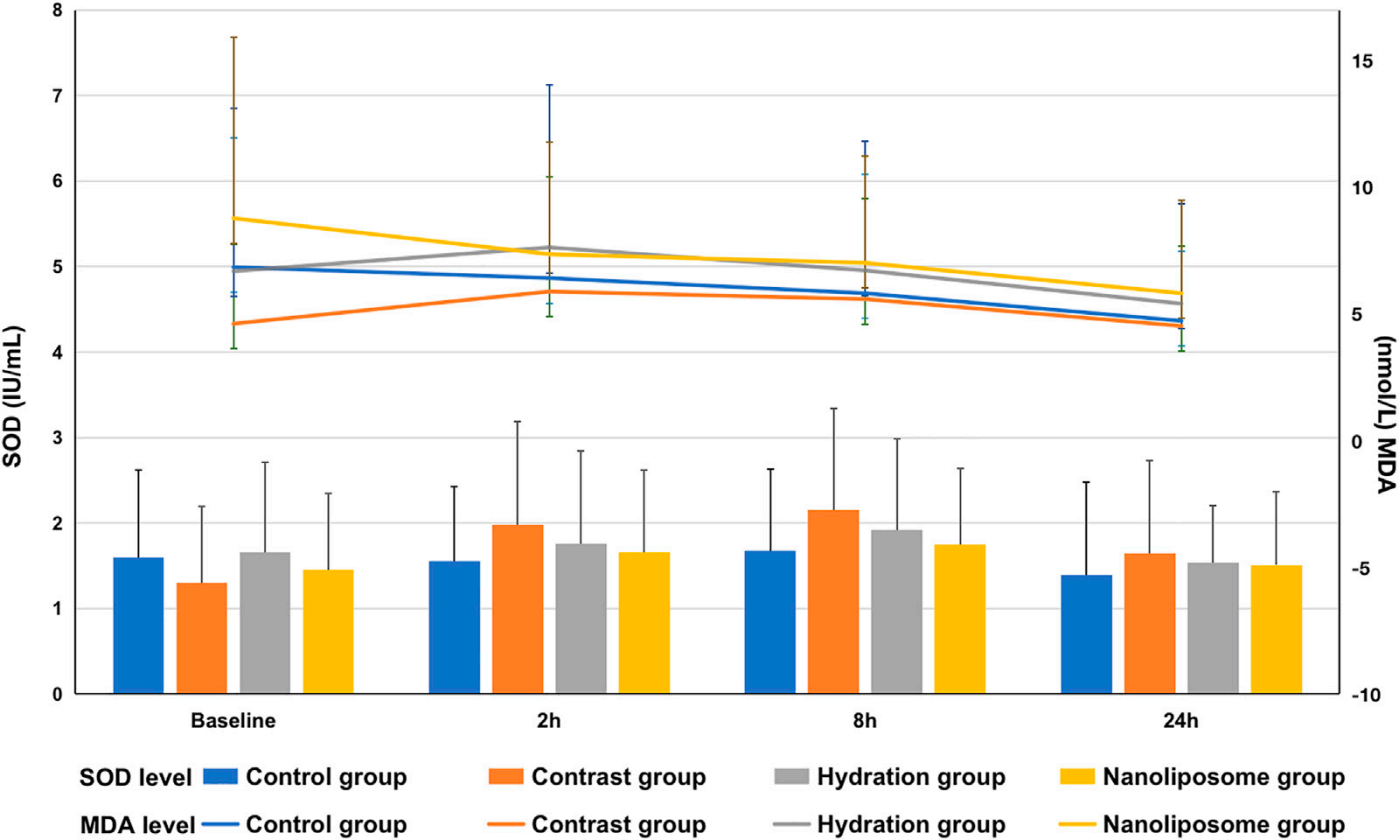
The contents of oxidative stress index SOD and MDA in four groups were detected by ELISA. SOD = superoxide dismutase; MDA=malondialdehyde. Values are expressed as mean ± SD. *Compared with the contrast group, *p*<0.05. **Compared with the contrast group, *p*<0.01. ^#^Compared with the hydration group, *p*<0.05. ^##^Compared with the hydration group, *p*<0.01.

Regarding oxidative stress markers like MDA and SOD, there were no significant differences between the four groups at 2, 8 or 24 h following contrast medium injection (Figure 6).

### The Lowest Contrast Induced Nephropathy Incidence Rate was Found in the Nano Liposome group

**FIGURE 7:**
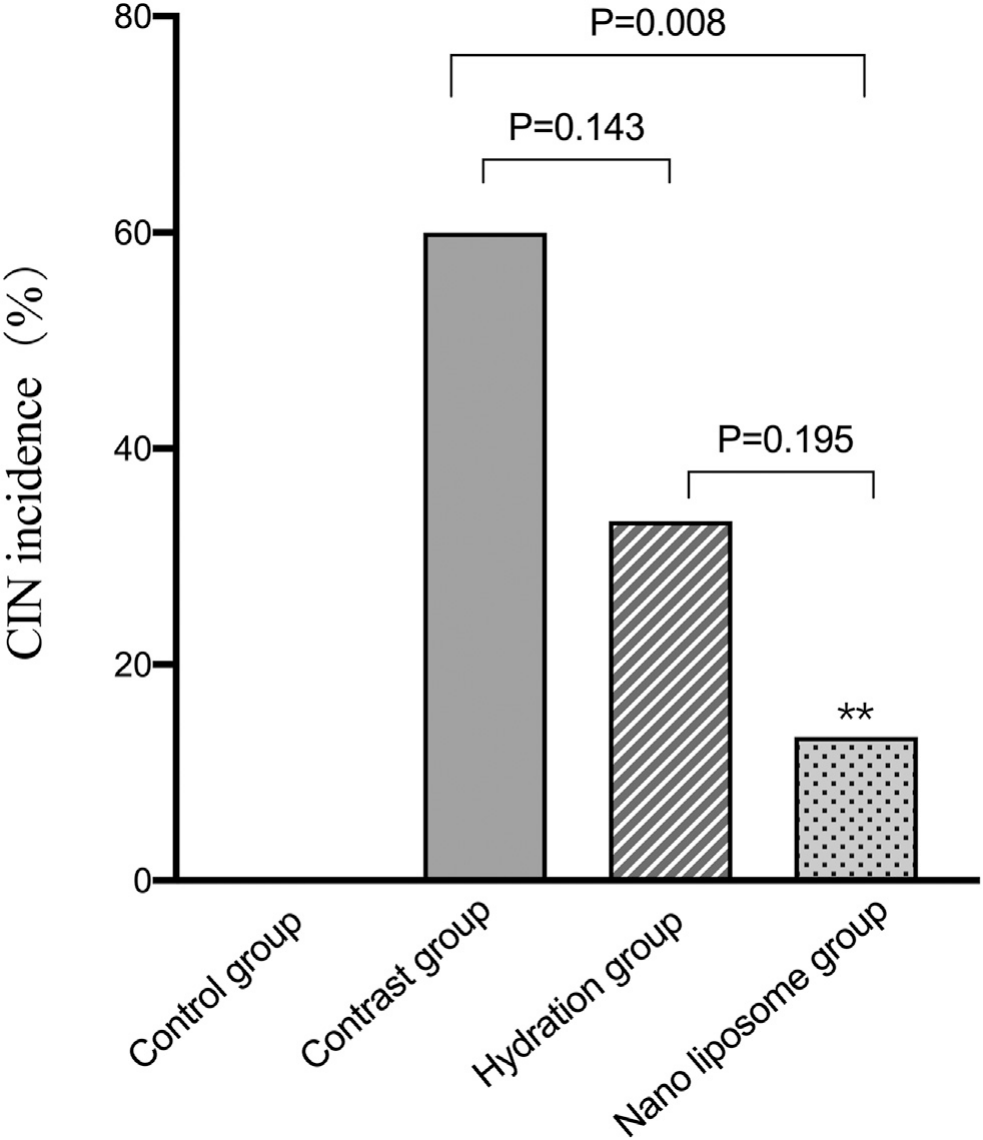
Quantitative comparison of CIN incidence in four groups. CIN = Contrast Induced Nephropathy. Values are expressed as mean ± SD. *Compared with the contrast group, *p*<0.05. **Compared with the contrast group, *p*<0.01. ^#^Compared with the hydration group, *p*<0.05. ^##^Compared with the hydration group, *p*<0.01.

The CIN incidence rates were as follows: control group (0/15), contrast group (9/15), hydration group (5/15), and nano liposome group (2/15). There were statistically significant differences between the following groups: contrast group (9/15) vs. control group (0/15), *p* = 0.002; contrast group (9/15) vs. nano liposome group (2/15), *p* = 0.008. The above values were statistically significant (*p* < 0.05). Although there were no significant differences between the hydration (5/15) and nano liposome groups (2/15) (*p* = 0.195), the incidence of CIN was lower in the nano liposome group. In addition, of the two intervention groups, only nano liposomes showed a statistically significant difference with the contrast group (Figure 7).

### Reverse Transcriptase-Polymerase Chain Reaction and Western Blot Analysis

**FIGURE 8:**
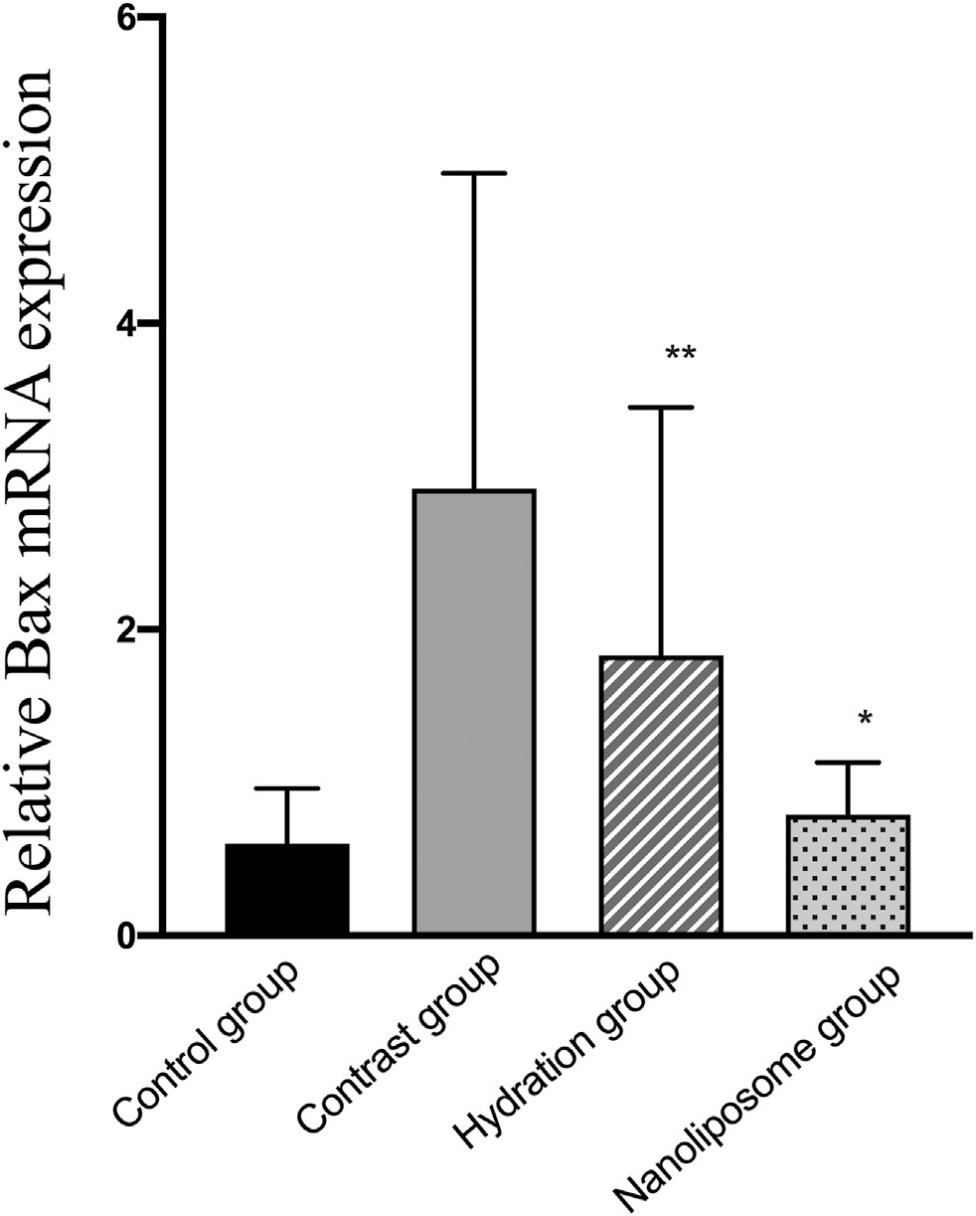
The relative mRNA expression of target gene was detected by Real-time PCR. Real-time PCR was used to validate the change in the expression of the Bax gene identified in four groups. Bax: Bcl-2 subfamily, pro-apoptotic factor. Values are expressed as mean ± SD. *Compared with the contrast group, *p*<0.05. **Compared with the contrast group, *p*<0.01. ^#^Compared with the hydration group, *p*<0.05. ^##^Compared with the hydration group, *p*<0.01.

**FIGURE 9:**
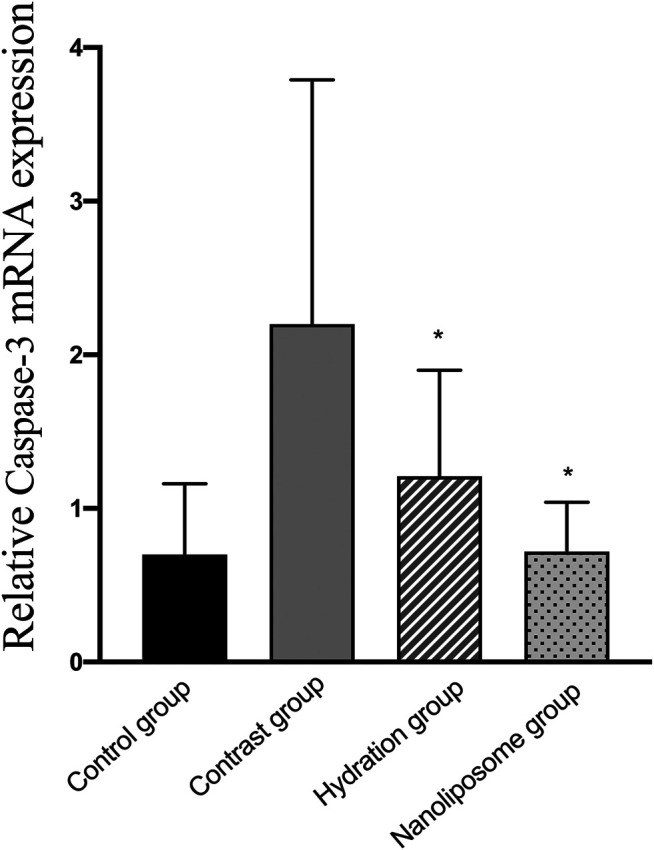
The relative mRNA expression of target gene was detected by Real-time PCR. Real-time PCR was used to validate the change in the expression of the Caspase-3 gene identified in four groups. Caspase-3 = cysteine aspartate specific protease 3. Values are expressed as mean ± SD. *Compared with the contrast group, *p*<0.05. **Compared with the contrast group, *p*<0.01. ^#^Compared with the hydration group, *p*<0.05. ^##^Compared with the hydration group, *p*<0.01.

**FIGURE 10:**
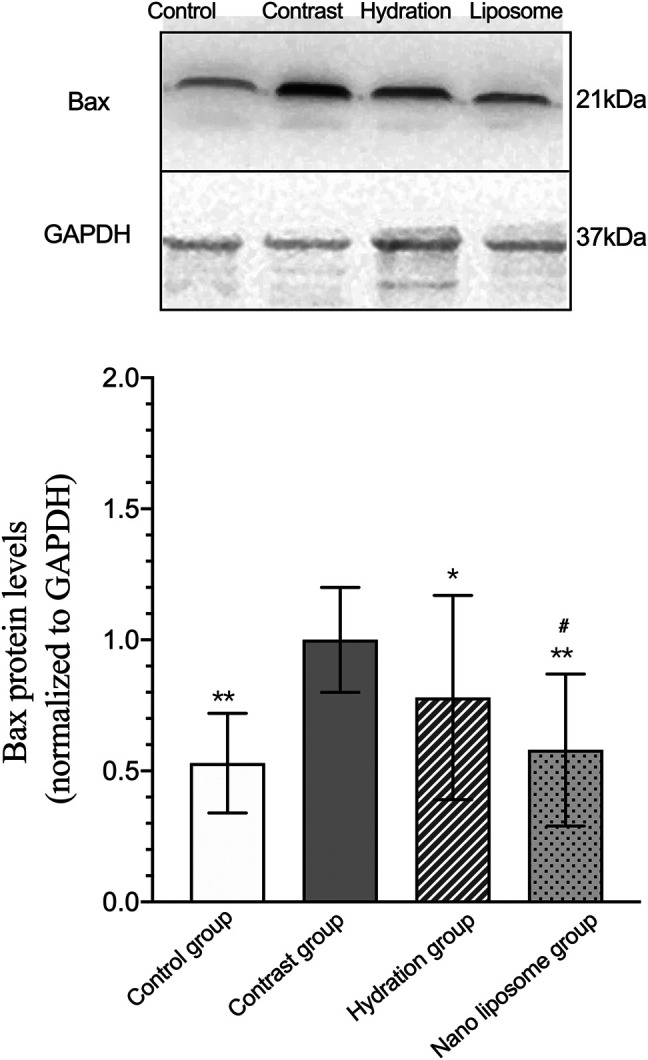
Representative western blot results and analysis of the Bax protein expression in the four groups. Bax: Bcl-2 subfamily, pro-apoptotic factor. Values are expressed as mean ± SD. *Compared with the contrast group, *p*<0.05. **Compared with the contrast group, *p*<0.01. ^#^Compared with the hydration group, *p*<0.05. ^##^Compared with the hydration group, *p*<0.01.

**FIGURE 11:**
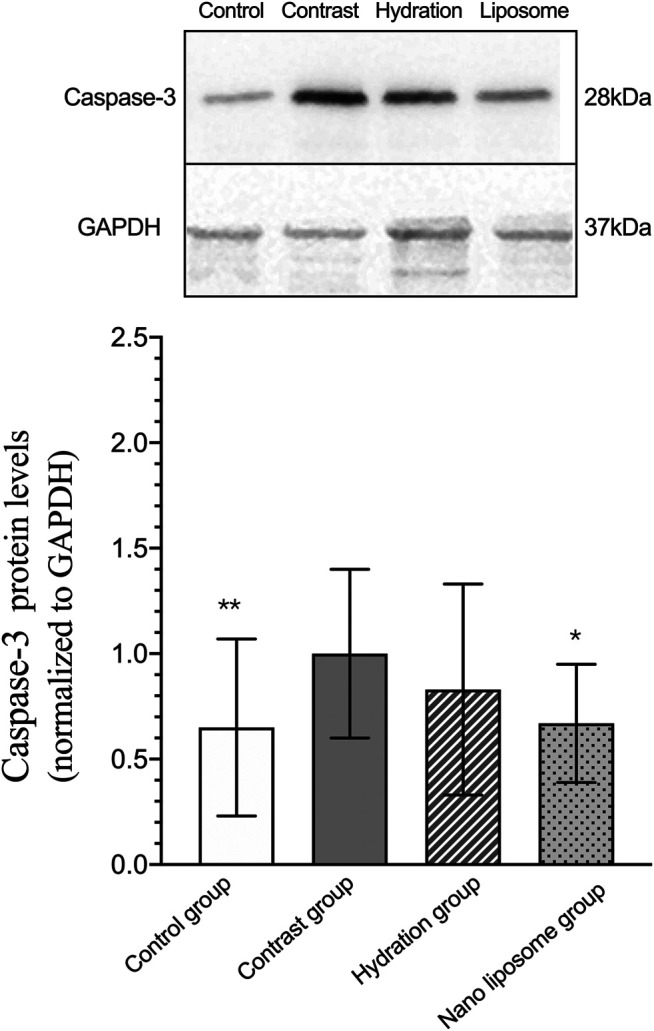
Representative western blot results and analysis of the Caspase-3 protein expression in the four groups. Caspase-3 = cysteine aspartate specific protease 3. Values are expressed as mean ± SD. *Compared with the contrast group, *p*<0.05. **Compared with the contrast group, *p*<0.01. ^#^Compared with the hydration group, *p*<0.05. ^##^Compared with the hydration group, *p*<0.01.

**FIGURE 12:**
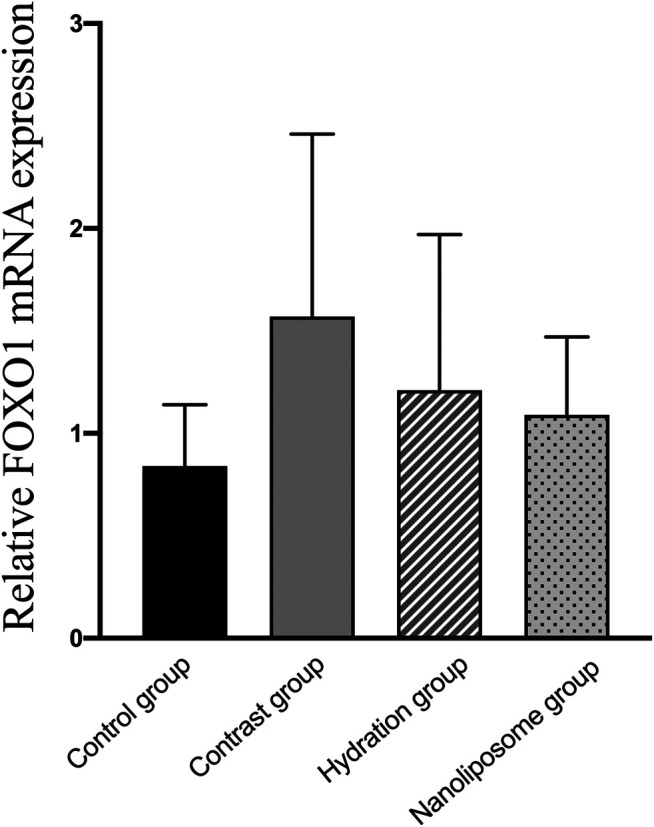
The relative mRNA expression of target gene was detected by Real-time PCR. Real-time PCR was used to validate the change in the expression of the FOXO1 gene identified in four groups. FOXO1 = Forkhead box O1. Values are expressed as mean ± SD. *Compared with the contrast group, *p*<0.05. **Compared with the contrast group, *p*<0.01. ^#^Compared with the hydration group, *p*<0.05. ^##^Compared with the hydration group, *p*<0.01.

**FIGURE 13:**
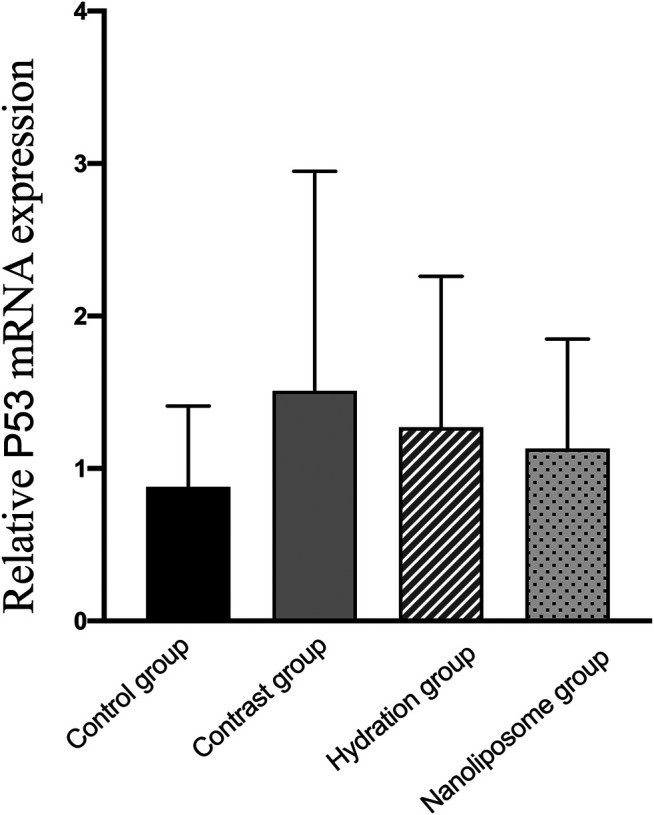
The relative mRNA expression of target gene was detected by Real-time PCR. Real-time PCR was used to validate the change in the expression of the P53 gene identified in four groups. P53: a apoptosis inducing factor. Values are expressed as mean ± SD. *Compared with the contrast group, *p*<0.05. **Compared with the contrast group, *p*<0.01. ^#^Compared with the hydration group, *p*<0.05. ^##^Compared with the hydration group, *p*<0.01.

Analysis of gene expression in the four transcriptomes was carried out using the log_2_ Ratio (FPKM) method. This analysis revealed differential expression of the following four genes: Bax, caspase-3, FOXO1, and P53. Only the Bax and caspase-3 genes were differentially expressed in the four groups. RT-PCR and Western blot analysis were used to validate these changes in gene expression. The results revealed similar trends in the expression of these two genes when compared with the sequencing results. Also, the relative expression of Bax and caspase-3 was significantly different in the four groups (*p* < 0.05). The relative expression of Bax and caspase-3 mRNA in the contrast group was higher than in the control group. Relative expression of Bax and caspase-3 mRNA in the hydration and nano liposome groups was lower than in the contrast group (*p* < 0.05) (Figures 8, 9). Although there were no significant differences between the two intervention groups (*p* > 0.05), relative expression of Bax and caspase-3 mRNA was lower in the nano liposome group. Similar changes were observed by Western blot. Compared with the contrast group, Bax protein expression in the nano liposome group was significantly decreased (*p* < 0.05). Compared with the hydration group, expression of Bax protein in the nano liposome group was lower, and this difference was statistically significant (*p* < 0.05). Regarding caspase-3 protein expression, there were no significant differences between the hydration and contrast groups, but expression in the nano liposome group was lower than in the contrast group, and this difference was statistically significant (*p* < 0.05) (Figures 10, 11). The results also showed that the relative expression of FOXO1 and P53 was no significantly different in the four groups (*p* > 0.05) (Figures 12, 13).

### Histological Changes Under the Optical and Electron Microscope

**FIGURE 14:**
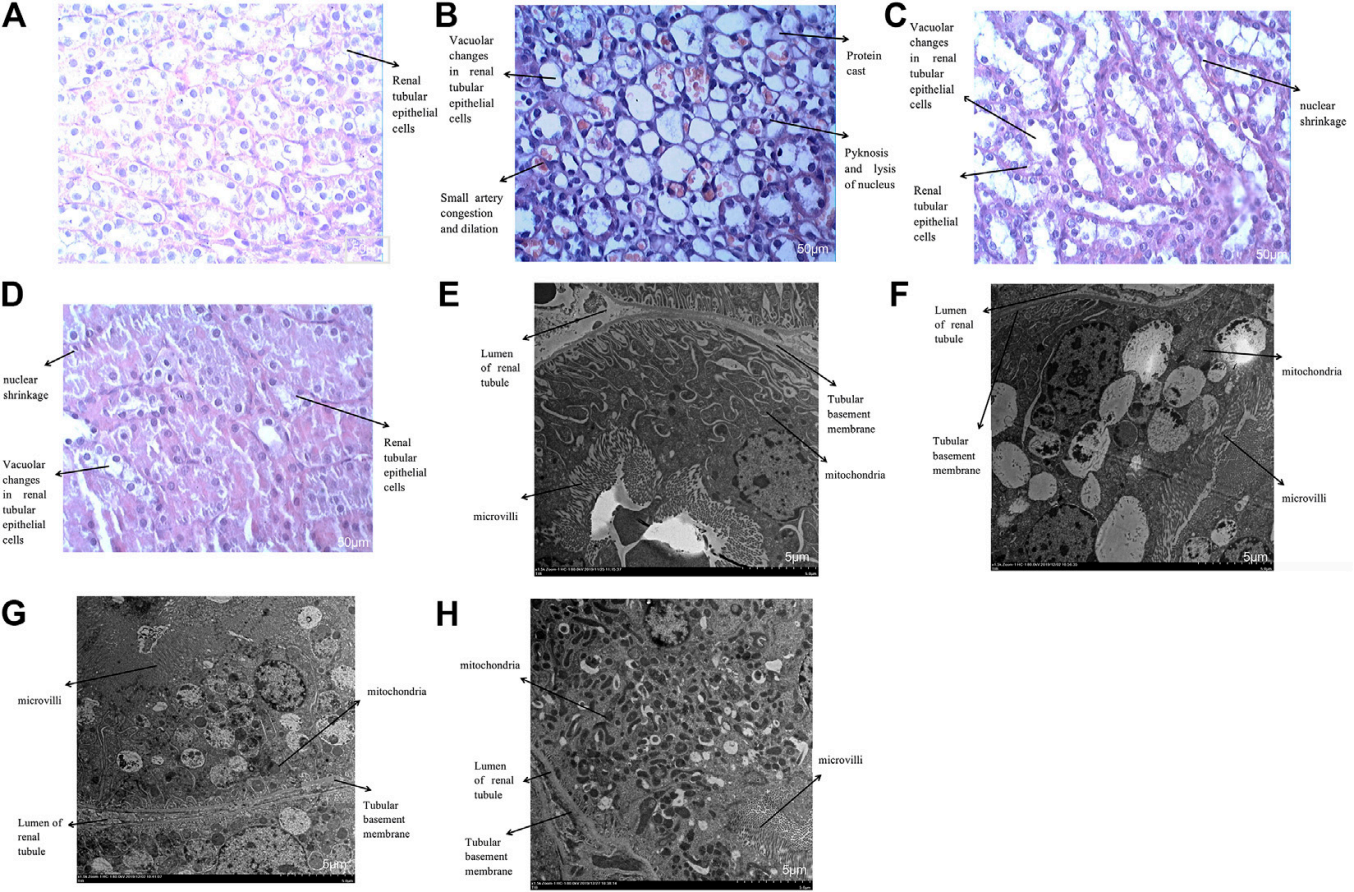
**(A–D)**: Histological changes of four groups under optical microscope (Enlarge the picture to 400x). **(A)**: Haematoxylin staining of rabbit kidney in the control group; **(B)**: Haematoxylin staining of rabbit kidney in the contrast group; **(C)**: Haematoxylin staining of rabbit kidney in the hydration group; **(D)**: Haematoxylin staining of rabbit kidney in the nano liposome group. **(E–H)**: Histological changes of four groups under electron microscope (Enlarge the picture to 1500 × 5 µm). **(E)**: Ultrastructure of rabbit kidney in the control group; **(F)**: Ultrastructure of rabbit kidney in the contrast group; **(G)**: Ultrastructure of rabbit kidney in the hydration group; **(H)**: Ultrastructure of rabbit kidney in the nano liposome group.

The histological changes under the optical microscope were as follows: in the control group, the renal tubular structure was basically normal; in the contrast group, vacuolar degeneration of renal tubular epithelial cells, abundant nuclear pyknosis, lysis, urinary casts, luminal occlusion, renal arteriole dilatation and congestion were observed, even the tubular structure disappeared; in the hydration group, abundant tubular epithelial cell vacuolar degeneration, nuclear pyknosis and renal arteriolar dilatation were observed; in the nano liposome group, a small number of tubular epithelial cells showed vacuolar degeneration and a small amount of nuclear pyknosis (Figures 14A–D). The four groups were also analyzed under the electron microscope. The ultrastructure in the control group was normal; the ultra structural changes in the contrast group included mitochondrial swelling in renal tubular epithelial cells, disorder of the ridge structure, which appeared broken and disappeared, microvilli inside the lumen and clearly visible apoptotic cells; in the hydration and nano liposome groups mitochondrial swelling in renal tubular epithelial cells was clearly visible, although the swelling was not as pronounced and the structure of the internal boundary ridge was normal, with few broken areas, plus few apoptotic cells, and no pyknotic nuclei. There was less apoptosis in the nano liposome group than in the hydration group (Figures 14E–H).

## Discussion

In this study, we found that nano liposome administration reduced the incidence of CIN in New Zealand rabbits and improved their renal function. The nano liposomes reduced renal ultrastructure changes, preventing damage by the contrast media.

Hydration therapy is currently the most widely used and inexpensive means of preventing CIN. Hydration works mainly by increasing the effective circulating blood volume through fluid supplementation, diluting the contrast agent, and increasing renal perfusion to achieve a diuretic effect. At the same time, hydration therapy can also inhibit the renin-angiotensin-aldosterone system (RAAS), increase NO, dilate blood vessels and reduce medulla ischemia. Hydration can also dilute the contrast agent inside the renal tubules, thereby greatly attenuating direct damage to renal tubular epithelial cells (Seeliger et al., 2010). However, since hydration therapy involves intravenous or oral administration of large amounts of fluid which increase the cardiac preload, it is likely to cause acute heart failure in patients with abnormal cardiac ejection fraction. Therefore, the rate and total dose should be carefully monitored (Stacul et al., 2011).

The effectiveness of hydration for the mitigation and prevention of CIN is unquestionable (Song et al., 2019). However, we found that the Scr value in the nano liposome group was lower than in the hydration group at 8 and 24 h, and the incidence of CIN in the nano liposome group was also significantly lower than in the hydration group. These differences were statistically significant. These results indicate that nano liposomes play an important preventive role against kidney injury in CIN, alleviating the degree of kidney damage and protecting renal function.

Our other experiments showed that the mechanism of action of nano liposomes involved reduction of apoptosis, or programmed cell death (Yang et al., 1995; Baptiste and Fehlings, 2006; Sayer et al., 2006). Apoptosis is a process under the control of many genes, including caspase family, Bcl-2 family and P53 genes (Oudega and Xu, 2006; Papastefanaki et al., 2007; Di Meo et al., 2020). Many genes which are highly homologous to Bcl-2 have been found and together they form a large Bcl-2 family. Some of them can inhibit apoptosis, while others can promote apoptosis. Bcl-2 is an anti-apoptotic protein, whereas Bax is one of the pro-apoptotic proteins (Berrieman et al., 2005; Song et al., 2012). Studies (Solà-Riera et al., 2020) have shown that Bcl-2 can enhance the resistance of cells to most forms of DNA damage and inhibit apoptosis induced by drugs, but it cannot inhibit the cell damage caused by these factors, nor can it promote DNA repair. The inhibition of apoptosis by Bcl-2 may be related to the following factors (Tator, 2006; Tsutsumi et al., 2006; Li et al., 2013): an anti-oxidative effect, as seen during the induction of cell death triggered by the removal of growth factors, where overexpression of Bcl-2 can reduce the production of oxygen free radicals; also, Bcl-2 can indirectly modulate apoptosis by regulating the concentration of intracellular calcium ions; finally, overexpression of Bcl-2 can inhibit changes in mitochondrial permeability, influence the formation of macropores, and thus inhibit apoptosis. Bax is the most important apoptosis gene and belongs to the Bcl-2 gene family. Bax protein can form a dimer with Bcl-2 and inhibit Bcl-2. Therefore, Bax is an important pro-apoptotic gene. Interaction between these two genes can regulate apoptosis. Other studies (Zhou et al., 2007) have shown that contrast media can induce apoptosis of renal cells by promoting the expression of Bax and decreasing Bcl-2 expression. The apoptosis of renal cells induced by contrast medium is dose- and time-dependent.

As mentioned previously, Bax is the most important pro-apoptotic gene in the human body, it promotes apoptosis by inducing the release of caspase proteins, such as caspase-3 (Raghupathi et al., 2003). Caspase-3 is considered the main terminal enzyme in the process of apoptosis and is an important mediator of the cell killing effect (Gong et al., 2019). Caspase-3 contains 277 amino acid residues and has a molecular weight of about 32 KD (Zhao et al., 2018). The main substrate of caspase-3 is polymerase, an enzyme involved in gene integrity monitoring and DNA damage repair. Caspase-3 can also cleave a novel PKC to activate it. Other experiments have shown that this novel PKC is involved in the induction of apoptosis and is an important component of the CTL cell killing mechanism (Yang et al., 2019).

Studies have shown that the Bcl-2/Bax ratio is the key factor which determines the degree of apoptosis (Rai et al., 2019). If the relative amount of Bax is higher than that of Bcl-2, the number of Bax homodimers will increase and promote cell death. In contrast, if the relative amount of Bcl-2 is higher than that of Bax, it will promote the formation of Bcl-2/Bax heterodimers, increase the number of Bcl-2 homodimers, and inhibit cell death. Higher Bcl-2/Bax ratios inhibit apoptosis, whereas lower Bcl-2/Bax ratios promote apoptosis (Chen et al., 2006).

Our results showed statistically significant differences in serum Bax and Bcl-2 levels between the four experimental groups of New Zealand rabbits 24 h after injection of contrast medium. In particular, Bax levels in the nano liposome group were lower than in the hydration and contrast groups, whereas Bcl-2 levels in the nano liposome group were higher than in the hydration and contrast groups, and these differences were statistically significant. This suggests that CIN can induce apoptosis by increasing the expression of pro-apoptotic proteins and decreasing the expression of anti-apoptotic proteins, whereas hydration and administration of nano liposomes can prevent this effect. Similarly, Bcl-2/Bax ratio is the important index which determines the degree of apoptosis. Our results indicate thoroughly that nano liposomes can prevent the occurrence of CIN by decreasing apoptosis caused to the kidney by the contrast agent, and this effect is stronger than that seen with hydration. About oxidative stress markers, such as SOD and MDA, we made statistical calculation, but there was no statistical difference among the groups. This indicates that nano liposomes can not prevent the occurrence of CIN by decreasing oxidative stress caused to the kidney by the contrast agent.

In our study, relative expression levels of Bax and caspase-3 were also measured by RT-PCR. The results showed that the relative expression of Bax and caspase-3 was significantly different in the four groups. Similar changes were observed by Western blot analysis. The only difference was that caspase-3 levels decreased significantly in the nano liposome group when compared with the contrast group, and this difference was statistically significant. Regarding Bax, levels of expression were significantly lower in the two intervention groups when compared with the contrast group, although the nano liposome group showed a better effect than the hydration group. These results, which are consistent with our previous ELISA results, indicate that apoptosis plays a decisive role in the development of CIN. Meanwhile, we also compared other anti-proliferation and anti-apoptosis markers in RT-PCR, such as FOXO1 and P53. There was no significant difference among the groups, but there was a trend of improvement in the nano liposome group. We speculate that nano liposomes can prevent CIN by decreasing proliferation and apoptosis caused to the kidney. However, there was no significant difference in this result.

Optical and electron microscopy examination of renal tissue in the contrast group showed severe damage, with vacuolar degeneration of renal tubular epithelial cells, nuclear pyknosis and lysis, mitochondrial swelling in renal tubular epithelial cells and disorder of the marginal ridge structure. The results of our study are basically consistent with those reported by others at home and abroad (Krzossok et al., 2011; Ozkan et al., 2012; Gong et al., 2013). Compared with the contrast group, the degree of renal injury in the nano liposome and hydration groups was significantly reduced, but the degree of renal injury in the nano liposome group was clearly less, indicating that nano liposome administration works better than hydration.

Considering the literature and our experimental results, nano liposomes can protect the stability of mitochondrial trans-membrane potential of renal cells by reducing the apoptosis injury of kidney and affecting the metabolism of mitochondria; at the same time, nano liposomes can directly fuse with the renal cell membrane, stabilize the function of the cell membrane, delay the apoptosis of organelles, and ultimately reduce the renal damage of New Zealand rabbits (Bertrand et al., 2010; Ibarguren et al., 2013). The results provide a new measure for the effective prevention of CIN in clinic, and also provide a theoretical basis for the development of new contrast agents. In the future, if nano liposomes can be applied to humans, it can effectively reduce the toxic effect of contrast agent on renal function, reduce the hospitalization time, save the hospitalization expenses, reduce the occurrence of cardiovascular and renal events after PCI, and improve the short-term and long-term prognosis of patients.

This study had a few limitations. In this paper, a relatively small number of animals, oxidative stress and apoptosis indexes were selected. In the later stage, we need to further explore the preventive mechanism of nano liposomes on CIN by more animals, more oxidative stress and apoptosis index experiments. Furthermore, We need to study *in vitro* in order to reveal the mechanism of CIN. Hence, additional studies should be performed for a longer period to measure these parameters.

## Conclusion

In conclusion, we developed a successful model of CIN in New Zealand rabbits, and compared the biochemical, PCR and Western Blot indexes, microscopic pathological changes in four key experimental groups: control, contrast, hydration and nano liposome. Our results showed that the occurrence of CIN and the mechanism of nephrotoxicity may be related to apoptosis. Nano liposomes can prevent the occurrence of CIN by decreasing apoptosis, reducing the damage caused to the kidney by the contrast agent, and this effect is stronger than that seen with hydration. These results suggest new therapeutic approaches for the prevention of CIN.

## Data Availability

The raw data supporting the conclusion of this article will be made available by the authors, without undue reservation.
